# Genome Sequence of *Dermacoccus* Strain Tok2021, a Soil Actinobacterium Isolated from a Pinus radiata Forest

**DOI:** 10.1128/mra.00844-21

**Published:** 2022-02-03

**Authors:** C. Armstrong, D. Sen, K. Walker, L. Garrett, A. Byers, S. Wakelin

**Affiliations:** a Scion, Riccarton, Christchurch, New Zealand; b Scion, Rotorua, New Zealand; Indiana University, Bloomington

## Abstract

*Dermacoccus* strain Tok2021 (*Actinobacteria*) is a soil bacterium, isolated from commercial Pinus radiata forest soil from Tokoiti, New Zealand. The bacterium has a draft genome size of 3,101,786 bp and harbors genes involved in antibiotic production, siderophore production, and N_2_ fixation.

## ANNOUNCEMENT

Pinus radiata D. Don is the most widely grown plantation tree species in New Zealand (1). *P. radiata* trees and forest ecosystems provide a range of ecosystem services, spanning carbon storage and regulation of water cycling through to supporting forest productivity and health (2). The delivery of these are all connected, either directly or indirectly, to the biology and functioning of the forest soil (3).

*Actinobacteria* is a ubiquitous bacterial phylum present in *P. radiata* soils, comprising up to 20% of the total community abundance (4). Yet the role of these bacteria (and most others) in forest soils and their contribution toward the delivery of ecosystem services is poorly described. In order to better enable the inference of function from community sequencing studies, the addition of annotated genomes of isolated species to reference databases is vital.

Toward this end, bacteria were isolated into a pure culture from soil collected from a recently clear-cut harvest *Pinus radiata* stand in Tokoiti Forest, Otago, New Zealand (approximate location, 46°10′9″S, 169°57′E). The soil sample was collected from the topsoil layer and is classified as a mottled fragic pallic soil in the New Zealand Classification System (5). A soil solution was made by mixing 1 g soil into 100 mL sterilized ddH_2_O for 4 h; this solution was then serially diluted to 10^−8 ^g soil^−1 ^mL in a biological safety cabinet. Aliquots of 100 μL were spread onto “forest floor agar,” and the plates were kept in the dark at room temperature until a single colony formed. Once a colony formed, the plate was sent for sequencing. Forest floor agar was a medium made by steeping 200 g of forest floor material (litter and other plant material) overnight in 300 mL water, centrifuging and filtering the supernatant, and adjusting the pH to 5.5 to 5.6. Reasoner’s 2A (R2A) agar was then added to a 10% forest floor medium and autoclaved.

Bacterial strain Tok2021 was sequenced at the Microbial Genome Sequencing Center (MiGS) (Pittsburgh, PA, USA). Genomic DNA was extracted using Qiagen blood and tissue kits, and libraries were prepared by tagmentation using the Illumina DNA prep kit (6). Illumina adaptors and barcodes were added via TruSeq primers S501 to S508 and N701 to N712 (6). Sequencing was performed on a NextSeq 550 platform and generated 2.5 million paired-end reads 150 bp long. All reads were demultiplexed using bcl2fastq software, which filters out reads with low quality (Q) scores (<Q30). The trimmed reads were assembled using Unicycler v0.4.8 with default parameters (7). Contigs less than 200 bp were removed and the quality of assembly was checked using QUAST (8) (http://cab.cc.spbu.ru/quast/).

**FIG 1:**
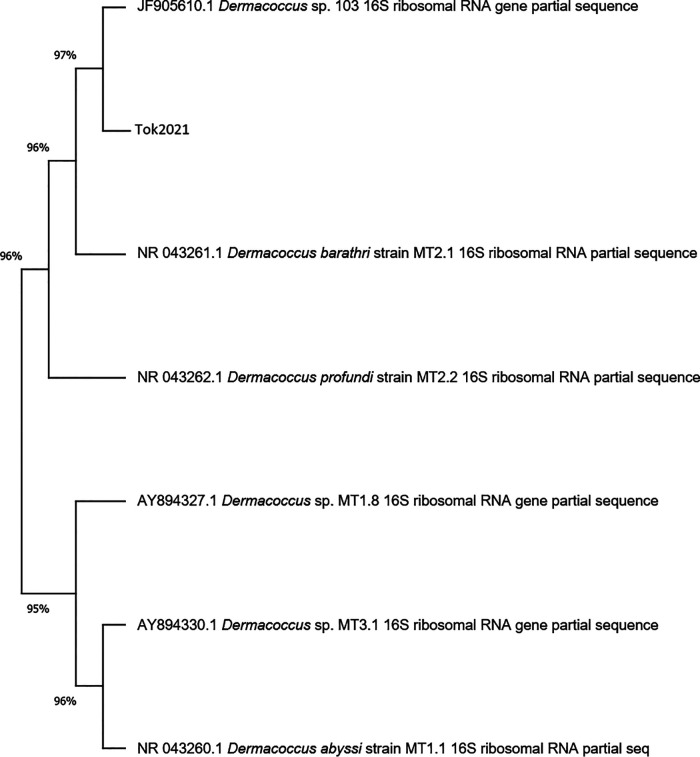
16S phylogeny of Tok2021 with the top 10 closest matched sequences from NCBI. The FASTA sequence files were aligned using MEGA with ClustalW.

The draft assembly has a length of approximately 3 Mbp with a sequencing depth of 125×, an *N*_50_ value of 109,593 bp, an *L*_50_ value of 10, and a G+C content of 68%. The total number of contigs generated was 49, with the largest scaffold being 436,392 bp long. Assignment of strain Tok2021 to *Dermacoccus* was based on the phylogenetic grouping of the full-length 16S rRNA gene to sequences in the NCBI, RDP, and SILVA databases. The NCBI phylogenetic tree (Fig. 1) was generated by aligning the top 10 matched 16S sequences from the NCBI database to Tok2021 using ClustalW in MEGA.

Genome annotation was carried out using Prokka v1.14.5 (9) and the NCBI Prokaryotic Genome Annotation Pipeline (PGAP) (10), resulting in 2,848 CDSs (protein-coding genes), 3 rRNAs, 50 tRNAs, and 1 transfer-messenger RNA (tmRNA). No CRISPR repeats were found. Of the CDSs, 74.3% are classified into at least one cluster of orthologous groups (COG) (11), and 84.51% are classified into at least one eggNOG group (12). Eight secondary metabolite clusters were predicted using antiSMASH (13) within MicroScope (14). One of these is a siderophore that is conserved among other members of *Actinobacteria*, and another is a cluster predicted to encode betalactone-class antibiotic compounds. The genome is also predicted to contain an N_2_-fixing gene, *nifH*, indicating that Tok2021 is a diazotroph.

### Data availability.

The whole-genome sequencing project has been deposited at DDBJ/ENA/GenBank under the accession number JAGTHU000000000. The raw reads can be accessed under the SRA accession number SRR14297439. The version described in this paper is version JAGTHU000000000.1.
